# Targeting Oncogenic Rewiring of Lipid Metabolism for Glioblastoma Treatment

**DOI:** 10.3390/ijms232213818

**Published:** 2022-11-10

**Authors:** Haksoo Lee, Dahye Kim, BuHyun Youn

**Affiliations:** 1 Department of Integrated Biological Science, Pusan National University, Busan 46241, Korea; 2 Department of Biological Sciences, Pusan National University, Busan 46241, Korea

**Keywords:** glioblastoma, lipid metabolism, cholesterol, fatty acid, sphingolipid, metabolic reprogramming

## Abstract

Glioblastoma (GBM) is the most malignant primary brain tumor. Despite increasing research on GBM treatment, the overall survival rate has not significantly improved over the last two decades. Although recent studies have focused on aberrant metabolism in GBM, there have been few advances in clinical application. Thus, it is important to understand the systemic metabolism to eradicate GBM. Together with the Warburg effect, lipid metabolism has emerged as necessary for GBM progression. GBM cells utilize lipid metabolism to acquire energy, membrane components, and signaling molecules for proliferation, survival, and response to the tumor microenvironment. In this review, we discuss fundamental cholesterol, fatty acid, and sphingolipid metabolism in the brain and the distinct metabolic alterations in GBM. In addition, we summarize various studies on the regulation of factors involved in lipid metabolism in GBM therapy. Focusing on the rewiring of lipid metabolism will be an alternative and effective therapeutic strategy for GBM treatment.

## 1. Introduction

Glioblastoma (GBM) is one of the most virulent brain tumors and is derived from glial cells [1]. The median survival of GBM patients is only 12–15 months with standard therapy, which includes surgery, chemotherapy with temozolomide (TMZ), and radiation [2,3]. In addition, patients with GBM have a 5-year survival rate of approximately 6.8% [4]. Regrettably, despite substantial studies and clinical applications, there has been no significant advance in ameliorating GBM treatment over time. As GBM has severe symptoms and surgical therapy is a delicate operation, many studies have focused on alternative and effective therapeutic strategies for GBM. Despite many efforts in GBM treatment, appropriate therapy for clinical situations is limited. Therefore, practical and applicable therapeutic strategies for GBM includ materials and equipment.

Cancer has peculiar metabolic properties for survival, and cancer metabolism is essential for maintaining a cancerous lifespan and harmonizing the tumor microenvironment [5]. For instance, cancer cells have a higher glycolytic metabolism and less oxidative phosphorylation than normal cells to avoid reactive oxygen species (ROS) and acquire cellular energy [6]. In addition, some cancers maintain decreased β-oxidation to survive in hypoxic environments by hypoxia-inducible factors [7]. Metabolic changes in cancer are related to chemo- or radio-immunoresistance [8]. Altered metabolism has been highlighted in GBM research to overcome the surgical limits and therapy resistance without side effects [9,10]. Specifically, reversing the Warburg effect elicits unique metabolic vulnerabilities [11] and increases oxidative stress [12].

Lipids, primary metabolites, are fundamental components of the cellular membrane and anabolic pathways in cells. In addition, energy storage, metabolism, and signaling pathways use lipids for cellular environmental responses. To maintain homeostasis, regulation of lipid metabolism is essential [13]. Recent studies have steadily researched aberrant lipid metabolism in GBM [14,15]. For instance, lipids are the primary cellular maintenance [16,17] and energy sources [18,19] in GBM. Blocking lipogenesis suppresses GBM growth [20] and targeting lipid peroxidation attenuates GBM [21,22]. As lipids are the main components of growth and alternative energy production pathways, lipid metabolism is an essential pathway for the growth and treatment resistance of GBM.

This review summarizes the normal lipid metabolism in the brain and abnormal metabolic signaling pathways in GBM. In addition, we have described recent studies on lipid metabolism-targeting treatments. Although GBM treatment has a long way to advance, the discussion and application of the distinct lipid metabolism of GBM will be a more effective and applicable therapeutic approach.

## 2. Reprogramming of Lipid Metabolism in GBM

### 2.1. Cholesterol Metabolism

#### 2.1.1. Cholesterol Metabolism in the Brain

Cholesterol in the brain is up to 25% of the total cholesterol level. In addition, cholesterol metabolism in the brain is different from other tissues because peripheral cholesterol cannot cross the blood–brain barrier (BBB) [23]. Cholesterol synthesis is complex. First, β-hydroxy β-methylglutaryl-CoA (HMG-CoA) converts acetyl-CoA to 3-hydroxy-3-methylglutaryl-CoA, which in turn converts 3-hydroxy-3-methylglutaryl-CoA to mevalonate. In the cholesterol synthesis pathway, HMG-CoA is a rate-limiting enzyme. Following mevalonate formation, mevalonate is converted into various intermediates to produce cholesterol [24]. Most brain cholesterol accumulates in the early developmental period, in which neurons are encircled by myelin. Moreover, the synthesis rate differed along with brain regions [25]. Existing cholesterol in the adult brain is maintained between 6 months and 5 years [26]. In contrast, plasma cholesterol is retained for a few days [27].

Cholesterol synthesis mainly occurs in the endoplasmic reticulum (ER), and de novo cholesterol is rapidly translocated from the ER to the cellular membrane [28]. The redistribution of cholesterol in different subcellular compartments is maintained by a combination of vesicle-mediated interorganelle transport and protein-mediated monomeric transfer through the aqueous cytoplasm. As cholesterol is insoluble in water, unintegrated cholesterol is located in the cytosol. Most cholesterol exists as a binding with proteins such as apolipoprotein E (Apo-E)-holding cholesterol particles. Although cholesterol transport is associated with these proteins, whether they have extra activation concerned with cholesterol than its transport activity remains unknown. The cholesterol synthesis and transport process are essential in the maintainence and functioning of cholesterol in the brain. Thus, abnormal synthesis and the transport of cholesterol cause aberrant cellular properties, including membrane composition, survival, and signaling pathways utilizing cholesterol and its intermediates.

#### 2.1.2. Cholesterol Metabolism Dysregulation in GBM

**Figure 1:**
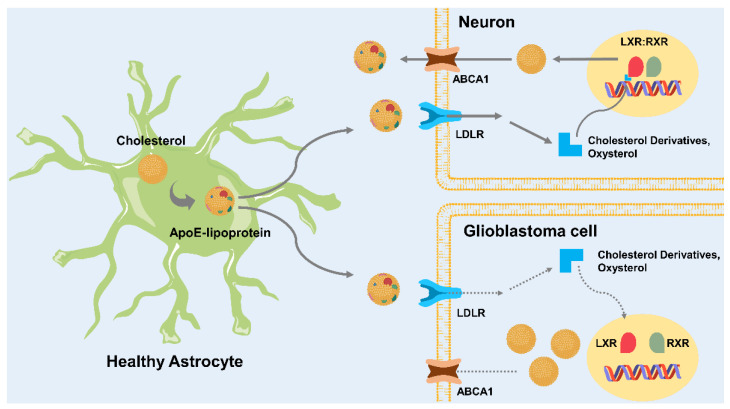
Neuron and GBM cells depend on astrocytes to synthesize cholesterol via de novo synthesis. Neurons and GBM cells do not use de novo synthesis of cholesterol. Instead, they uptake exogenous cholesterol in ApoE-lipoprotein from astrocytes. ApoE-lipoprotein interacts with LDL receptors. Then, oxysterol and cholesterol derivatives, which are physiological agonists for LXR, are produced in neurons. The activation of LXR results in its dimerization with RXR (LXR:RXR). Following the formation of LXR:RXR heterodimerization, ABCA1, an exporter of cholesterol as ApoE-lipoprotein, is increased. LXR:RXR decreases LDL receptor. Both the regulation of ABCA1 and LDL receptor can regulate the level of cellular cholesterol. In GBM cells, this cholesterol regulation is disrupted. Oxysterol and cholesterol derivatives are unable to activate LXR. Thus, the accumulation of cellular cholesterol in GBM. ABCA1, ATP-binding cassette transporter A1; ApoE, apolipoprotein E; LDLR, low-density lipoprotein receptor; LXR, liver X receptors; RXR, retinoid X receptors.

As cholesterol synthesis processes in the brain, astrocytes mainly synthesize cholesterol. Following cholesterol synthesis, it is transported by high-density lipoproteins containing Apo-E [29]. However, the metabolic necessity of GBM cells is primarily filled by exogenous cholesterol [23]. Thus, HMG-CoA reductase inhibitors, which inhibit cholesterol synthesis, could not affect GBM cells [23]. Liver X receptors (LXRα and LXRβ), which regulate cholesterol homeostasis, are essential to lipoprotein uptake in the brain [23]. The heterodimerization of LXR and retinoid X receptors (RXR) follows the transcriptional activation of genes associated with lipid transport between neurons and glial cells. Oxysterols, synthesized from cholesterol, act as endogenous LXR. The activation of LXR decreases cellular cholesterol in neurons and healthy astrocytes via increasing ATP-binding cassette subfamily a member 1 (ABCA1) sterol transporter and LDLR degradation. In GBM cells, endogenous LXR, which promotes cholesterol uptake, is not sufficiently produced. The LDL uptake and levels of LDL receptors were increased, and LXR ligands were decreased in GBM cells compared to in normal astrocytes (Figure 1) [23].

Cholesterol homeostasis is important for adaptation to changing environments because cholesterol maintains appropriate cellular membrane plasticity for the environment. A previous study reported that cholesterol uptake is associated with GBM growth and survival [23]. Therefore, abnormal synthesis and signaling of cholesterol cause cancer, thus suggesting a possible treatment target [30]. In addition, the higher levels of lipids in GBM cells are associated with epidermal growth factor receptor (EGFR)/PI3K/Akt. This pathway upregulates intracellular lipids and increases sterol regulatory element-binding protein-1 (SREBP-1), which increases lipid uptake in GBM [31]. These suggest that supplementing exogenous LXR and cholesterol homeostasis may represent a potential new anticancer strategy for GBM.

### 2.2. Fatty Acid Metabolism

#### 2.2.1. Fatty Acid Metabolism in the Brain

Lipids participate in broad cellular signaling processes. Specifically, fatty acids (FAs) have been well-characterized as drivers of intracellular signaling processes, including inositide phospholipid and cyclic AMP pathway [32]. FAs are classified as saturated fatty acids and unsaturated fatty acids according saturation state. There are one or more double carbon–carbon bonds in unsaturated fatty acids. Polyunsaturated fatty acids (PUFAs) have more than one carbon double-bond and well-defined roles. A previous study, reported that PUFAs are associated with neuronal signaling processes, which regulate neurotransmission, cellular survival, glucose homeostasis, mood, and cognitive function [33]. In addition, FAs are utilized for energy production. Because the brain is a highly energy-consuming organ, continuous supplementation of metabolites is critical for maintaining organ function. Energy necessity is mainly relieved by glucose metabolism. However, in a previous study, FA oxidation satisfied up to 20% of the energy requirements of astrocytes [34]. Utilizing FA for energy production occurs through the β-oxidation in mitochondria. In beta-oxidation, acyl-CoA synthase first converts FAs to fatty acyl-CoA (FA-CoA) [35]. Once this reaction occurs, carnitine palmitoyltransferase (CPT) transports substrates across mitochondrial membranes to the mitochondrial matrix [36]. FA-CoA produced by acyl-CoA synthase is converted into fatty acylcarnitine by CPT1. Next, CPT1 transported fatty acylcarnitine into the mitochondrial intermembrane space. CPT1 is the rate-limiting step in FA oxidation and is regulated by malonyl-CoA. As FA-CoAs can be directly utilized for oxidation or formation of glycerophospholipids, an enzymatic function of CPT1 is essential. Then, fatty acylcarnitine is transported into mitochondrial cytosol by acylcarnitine transferase through exchanges with free carnitines. In turn, CPT2 transforms FA-CoA. Through these reactions, FA-CoA can be utilized for the β-oxidation pathway [37]. After FA-CoA entereds the β-oxidation pathway, acyl-CoA dehydrogenase, enoyl-CoA hydratase, hydroxyacyl-CoA dehydrogenase, and ketoacyl-CoA thiolase act as a player of β-oxidation [35]. The total amount of enzymatic reaction cycle is FADH_2_, NADH, acetyl CoA, and fatty acyl derivative. FADH_2_ and NADH are directly utilized in the electron transport chain, and acetyl CoA enters the tricarboxylic acid cycle for energy production. As mentioned above, FAs are essential to the cellular signaling pathway and energy production in the brain. As FAs are necessary for normal cellular function, the alterations inFAs metabolism may cause disease and be a potential target.

#### 2.2.2. Fatty Acid Metabolism Dysregulation in GBM

Previous studies reported a correlation between FAs and GBM. Monounsaturated FAs (MUFAs) increase lipid droplet formation and FA oxidation in GBM. In addition, MUFAs are also associated with increasing glycolysis and proliferation [38,39]. Astrocytes are involved in the formation of lipid droplets that protect neurons in the face of high stress. Studies have shown that stressed neurons induce the formation of lipid droplets by migrating oxidized FAs to adjacent astrocytes, and the ability of GBMs to synthesize lipid droplets may suggest an astroglial property [40]. Metabolic profiling comparing low-grade astrocytoma and patient-derived GBM revealed that catabolism of FA is more than FA synthesis in GBM, suggesting that β-oxidation is the critical point for malignant GBM [41]. However, β-oxidation has both anabolic and catabolic roles, it may provide metabolic plasticity in GBM for adaptation to the harsh microenvironment. FA-CoA can enter the triacylglycerol (TAG) synthesis pathway through chain reactions catalyzed by glycerol-3-phosphate acyltransferase (GPAT), acylglycerophosphate acyltransferase (AGPAT), phosphatidic acid phosphohydrolase (lipin or PAP), and diacylglycerol acyltransferase (DGAT). Then, TAG is stored in a lipid droplet and utilized as an energy source by specific lipases [42]. Adipose triglyceride lipase (ATGL), hormone-sensitive lipase (HSL), and monoacylglycerol lipase (MAGL) mediate the hydrolysis of TAG [43]. Increased de novo FA biosynthesis is a hallmark of cancer [44], and is responsible for FA synthesis and upregulated in GBM. For example, increased FASN expression leads to high de novo FA synthesis levels, which are sufficient to promote movement and wound repair in glioma cells [45]. The treatment of TMZ with metformin decreases the level of FASN in an orthotopic GBM mouse model [46]. In addition, the expression of ATP citrate lyase (ACLY) is significantly upregulated in GBM. The inhibition of ACLY activity or expression results in a decreased growth in GBM [47].

**Figure 2:**
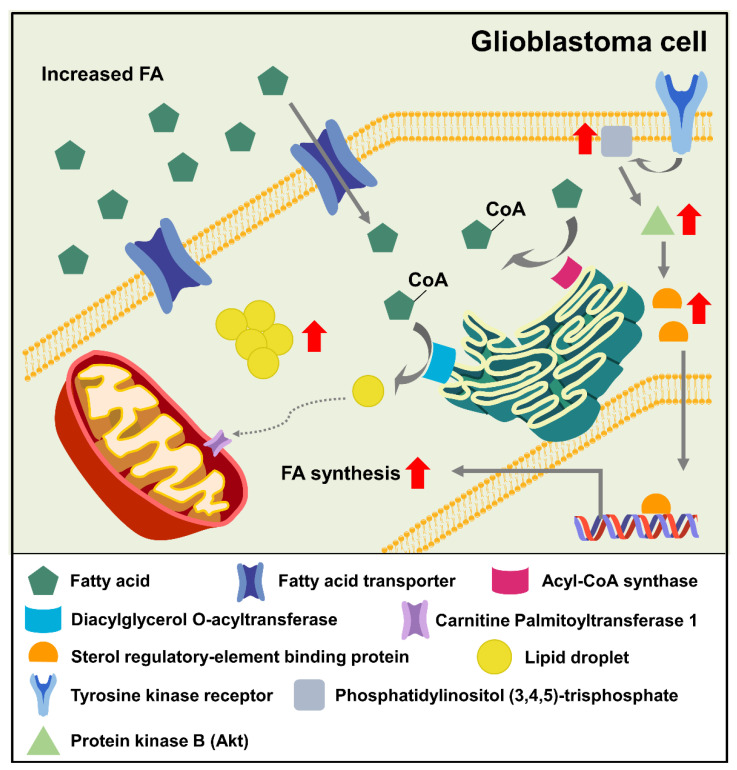
Alteration of FA metabolism in GBM. Fatty acid enters the cytosol through fatty acid transporter. Then, Acyl-CoA synthase converts FA to FA-CoA. FA-CoA is stored as lipid droplet through DGAT. GBM accumulates lipid droplets not utilized for fatty acid oxidation compared with normal cells. Together with this, SREBP is upregulated and activated in GBM by activating the PI3K/Akt pathway. Activated SREBP enters the nucleus, increasing transcription of FA synthesis-associated genes, including FASN, and ACLY. Thus, the level of FA is increased in GBM cells.

The upregulation of FA synthesis is likely due to the increased expression of the crucial transcriptional regulator SREBP in GBM cells. Oxygen and nutrient limitations are standard features of the tumor microenvironment and are associated with cancer progression and induction of metastasis [5]. The activation of SREBP is involved in FA and cholesterol metabolism under hypoxia [48]. In addition, SREBP1 is a downstream target of tumor-suppressor pathways, including the liver kinase b1, AMP-activated protein kinase (LKB-AMPK), and AKT pathways. Specifically, the phosphorylation of SREBP1 is induced by AMPK, resulting in the inhibition of activity and tumor growth. On the other hand, AMPK also phosphorylates acetyl-CoA carboxylase (ACC), inhibiting FA synthesis [49]. In GBM cells, AMPK activation increases ACC activity and its level [50]. The phosphoinositide 3-kinase/AKT signaling pathway is also activated in cancers [51]. Activation of this signaling pathway increases the expression of SREBP1 and cholesterol and FA synthesis-associated genes [52]. In addition, PI3K hyperactivation and EGFR mutations induce GBM growth and survival by activating SREBP-1 (Figure 2) [53]. These data indicate that the inhibition of SREBP activity may be a promising therapeutic strategy. Collectively, GBM utilizes FAs for energy production and signaling pathway response to hypoxic conditions and the requirement of nutrients. Thus, GBM-specific FAs metabolism may be a vulnerable target through inhibiting essential energy needs and signaling.

### 2.3. Sphingolipid Metabolism

#### 2.3.1. Sphingolipid Metabolism in the Brain

Sphingolipids, integral structural components of cell membranes, act as various signaling molecules to determine cell fate and function. Sphingolipids are composed of a long chain of sphingoid bases linked to FAs [54]. Serine palmitoyltransferase synthesizes the de novo synthesis of sphingolipids using serine and palmitoyl-CoA. Then, ceramide synthases generate ceramide from sphingolipid [55,56]. Following ceramide generation, ceramide is converted to various factors by specific enzymes. For instance, sphingomyelin, an essential component of myelin in the central nervous system (CNS), is transformed from ceramide by sphingomyelinases (SMases) [57]. Additionally, glycosphingolipids, which are also in the CNS, are formed from ceramide by glucosylceramide synthase, or ceramidases can process sphingosine. Sphingosine kinase 1 (SK1) or sphingosine kinase 2 (SK2) phosphorylate sphingosine to form sphingosine 1-phosphate (S1P), and S1P phosphatase (SPP) reverses this phosphorylation [56,58]. Glycosphingolipids are vastly enriched in the brain and related to the composition of cellular membranes, myelin sheaths of nerve axons, and cell signaling [59,60,61]. Glycosphingolipids are also associated with the differentiation of embryonic stem cells and neural stem cells through metabolic processes, resulting in the expression of sialic acid-containing glycosphingolipids, also known as gangliosides, in the neuronal membranes [62,63,64]. Sphingolipid consists of the cellular membrane and myelin sheath in the brain and stem cells. Thus, sphingolipid metabolism is necessary to intact cellular function in the brain, and deregulated sphingolipid metabolism may be a potential driver of disease, specifically cancer.

#### 2.3.2. Sphingolipid Metabolism Dysregulation in GBM

To reinforce its malignancy and capacity to survive, GBM has broad, subtle strategies, including aberrant sphingolipid metabolism. Specifically, ceramide is downregulated in GBM. A previous study reported that the ceramide level is lower in GBM tissue than in normal brain tissue. In addition, ceramide level in GBMs is directly related to histological grade and survival [65]. Not only is histological grade related to total ceramide, but S1P level is as well. S1P level is increased approximately nine-fold in GBM tissues compared to normal gray matter [66]. In addition, ceramide has a heterogeneous structure, as FAs with different acyl chain lengths, double bonds, and hydroxylations [67]. For this reason, ceramide depends on different fatty acyl precursors and specific enzymes, which produce different ceramide species [68]. As the result of liquid chromatography tandem mass spectrometry, there are differences in the distribution of fatty acyl chains of ceramides between different glioma cell lines [69], suggesting that different GBMs have broad species of ceramide. Specifically, the reduction in C18 ceramide was shown in human gliomas, which is related to malignancy grade. However, despite the heterogeneity of ceramide, C18 ceramide was decreased in different GBMs [66]. The level of C18 ceramide was reduced and was inversely correlated with metastasis in head and neck squamous cell carcinoma [70], suggesting that decreased C18 ceramide may contribute to GBM malignancy. In addition, human GBM has a significantly low level of sphingomyelin than non-tumor cells [71]. Decreased sphingomyelin is related to GBM tumorigenic transformation [72].

**Figure 3:**
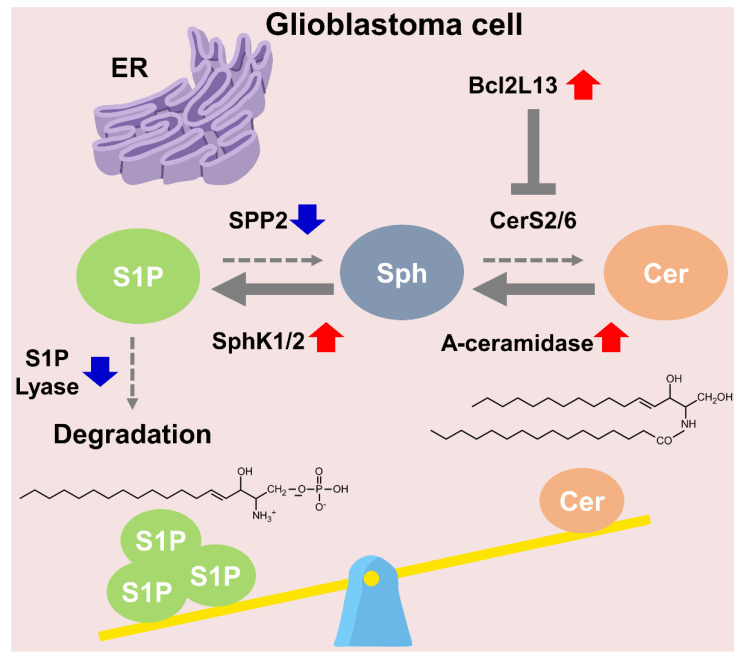
Multiple dysregulations in sphingolipid metabolism occur in GBM and disrupt S1P/ceramide balance in favor of S1P. In GBM cells, A-ceramidase, SphK1/2, and Bcl2L13 are upregulated. A-ceramidase converts ceramide to sphingosine, and SphK1/2 converts sphingosine to S1P. Bcl2L13 inhibits the function of CerS2/6. Together with is, SPP2 and S1P lyase are downregulated in GBM cells. The degradation of S1P via S1P lyase and conversion of S1P to sphingosine are decreased in GBM cells. Thus, S1P is accumulated in GBM. S1P, sphingosine 1-phosphate; SPP2, S1P phosphatase; Sph, sphingosine; Cer, ceramide; CerS, ceramide synthase; SphK, sphingosine kinase; Bcl2L13, B-cell lymphoma 2-like 13; A-ceramidase, acid-ceramidase.

Previous studies showed molecular explanations for the abnormal distribution of ceramide and S1P in GBM. Acid ceramidase, which produces sphingosine from ceramide, was significantly increased in GBM [66]. Moreover, B-cell lymphoma 2-like 13 (Bcl2L13), an uncommon member of the Bcl-2 family, is overexpressed in GBM. Bcl2L13 acts as a ceramide synthase (CerS) inhibitor [73]. Interestingly, as Bcl2L13 inhibits the activity of CerS2 and CerS6, Bcl2L13 functions as an anti-apoptotic protein by protecting mitochondrial membrane integrity. As sphingosine kinases K1 and K2 (SphK1 and SphK2) are upregulated, S1P is increased in GBM [74,75,76]. In addition, with SphK upregulation, SPP2, which is localized in ER and functions in S1P, is lower in GBM than normal gray matter [66,77]. Notably, SPP1 increases ceramide levels in the ER via recycling sphingosine [78], suggesting that decreased SPP2 levels in GBM increase S1P and induce ceramide levels. Together with this, it is notable that the S1P lyase-related gene is deleted in human GBMs [79]. Thus, as GBM adapts to downregulate ceramide and upregulate S1P to induce reprogramming in sphingolipid metabolism, GBM has more aggressiveness and defense against death. Using a mathematical model to determine how sphingolipid metabolism is altered in GBM cells, there is a significant difference in that sphingolipid is preferentially into S1P synthesis in GBM cells. In contrast, sphingosine is mainly recycled into ceramide in normal astrocytes [80]. S1P exerts multiple roles through its five specific receptors (S1P 1–5) [81], and S1P 1–3 and S1P 5 receptors were found in human GBM [82,83]. In a recent study, the mRNAs of S1Ps were increased in human GBM compared to normal brains, with increasing malignancy (Figure 3) [76]. Collectively, the aberration of sphingolipid metabolism and alteration of sphingolipid are noticeable points in GBM treatment. For further GBM treatment, sphingolipid-related metabolic alterations and dysfunction are needed for GBM research.

## 3. GBM Therapy Targeting Lipid Metabolism

As in the previous section, lipid metabolism is dysregulated in GBM. GBM cells accumulate cholesterol by activating cholesterol uptake and inhibiting cholesterol excretion. Cellular cholesterol is utilized for GBM growth and survival. In addition, increased cholesterol activates oncogenic signaling. GBM also increases FA synthesis and accumulation of lipid droplets. As regulating FA synthesis and lipid droplets, GBM utilizes β-oxidation according to the situation. In addition, increased oncogenic signaling induces SREBP activation, and SREBP upregulates the expression of FA synthesis-related genes. The dysregulation of FA allows for GBM to adopt the appropriate strategy in a harsh microenvironment. The alteration of ceramide and S1P is also related to GBM aggressiveness and histological grade. Thus, targeting the lipid metabolism of GBM is a promising therapeutic target, considering the changed factors. Although the clinical application of lipid metabolism remains challenging, some previous studies reported promising therapeutic strategies targeting lipid metabolism in GBM. Targeting cholesterol-, FA-, and sphingolipid-related studies have been described in this section (Table 1).

### 3.1. Targeting Cholesterol Metabolism in GBM

A previous study reported that the ApoE peptide [(LRKLRKRLL)2C] specifically binds to LDLRs and penetrates the BBB, resulting in GBM-targeting therapy in vivo [84]. The other target is the cytochrome P450 family 46 subfamily A member 1 (CYP46A1). CYP46A1 catalyzes the conversion of cholesterol to 24S-hydroxycholesterol. CYP46A1 expression was significantly lower in GBM samples than in normal brain tissue. A reduction in CYP46A1 expression is associated with increased tumor grade and poor prognosis in human gliomas. Efavirenz, an activator of CYP46A1 that penetrates the blood–brain barrier, inhibits GBM growth in vivo [85]. In other cases, the combination of LXR623 and gamitrinib reduced tumor growth and induced cell death in GBM. These effects of LXR623 and gamitrinib are reversed by exogenous cholesterol [86]. In addition, sterol O-acyltransferase (SOAT1) is upregulated in GBM and controls cholesterol esterification and storage in GBM. Targeting SOAT1 inhibits GBM growth and increases survival in mouse models by inhibiting SREBP-1-regulated lipid synthesis [20]. Finally, the pharmacological inhibition of SREBP by 25-HC, fatostatin, and FGH10019 decreased SREBP-1 and SREBP-2 targeting genes and reduced the growth of the GBM cell line [87].

### 3.2. Targeting Fatty Acid Metabolism in GBM

A previous study reported that the mitochondrial FAO enzymes (CPT1A, CPT2, and ACAD9) and CD47 are related to recurrent GBM patients with poor prognosis. Etomoxir combined with anti-CD47 antibody sensitized radiotherapy and boosted phagocytosis via macrophage in recurrent GBM [88]. Medium-chain acyl-CoA dehydrogenase (MCAD) has been reported to be involved in lipid peroxidation. MCAD oxidizes medium-chain fatty acids (MCFA) and is upregulated in GBM. Depleting MCAD induced harmful metabolic changes, including accumulation of MCFAs, which increased lipid peroxidation, oxidative damage, mitochondrial damage, and apoptosis [22]. Another target of lipid peroxidation, DGAT1, has been previously reported. As in the case of MCAD, DGAT1 is upregulated in GBM to store FAs in TAG and lipid droplets. The inhibition of DGAT1 induced unbalancing of lipid homeostasis and increased β-oxidation, leading to excess reactive oxygen species, mitochondrial damage, cytochrome c release, and apoptosis [21].

### 3.3. Targeting Sphingolipid Metabolism in GBM

A previous study reported that sphingomyelin phosphodiesterase 1 (SMPD1), which converts sphingomyelin to ceramide, is a druggable target for GBM. The antidepressant fluoxetine could inhibit SMPD1 activity, inducing GBM death by inhibiting EGFR signaling and activating lysosomal stress [89]. In another SMPD1-related study, pimozide and loperamide inhibited the SMPD1 activity and promoted the induction of lysosomal membrane permeabilization (LMP) and the release of cathepsin B (CTSB) into the cytosol. Thus, pimozide and loperamide induce autophagy and lipotoxicity, resulting in LMP and GBM death [90]. N-acylsphingosine amidohydrolase 1 (ASAH1), which hydrolyzes ceramides, has been identified in other cases. ASAH1 is upregulated in GBM compared to non-tumor. Carmofur, and the inhibition of ASAH1, decreased in vitro migration of GBM cells and patient-derived xenograft models [91]. Finally, it has been reported that disialoganglioside GD2 is expressed in malignant gliomas. GD2 targeting by chimeric anti-GD2 dinutuximab beta is an available inhibitor against GBM [92].

## 4. Conclusions

Although further research is in progress, GBM remains an obstinate type of brain tumor. Metabolic reprogramming of GBM cells is one of the properties of growth and survival in unfavorable environments. Lipid metabolism in GBM is also essential for cellular growth, energy requirements, and oncogenic signaling. Anabolic and catabolic changes in lipid metabolism are inescapable and noticeable when alleviating GBM. However, focusing on the use of lipid metabolism to fully cure GBM has not been investigated because the physiological functions of lipid and lipid-related pathways in the brain and GBM are poorly understood. Research based on various alterations in lipid metabolism in GBM will be more compelling for GBM treatment. Thus, future investigations of therapeutic strategies focusing on lipid metabolism may provide new and practical concepts for GBM therapy.

## Figures and Tables

**Table 1:** Therapeutic strategies targeting lipid metabolism in GBM.

Lipids	Target	Regulation	Method
Cholesterol	Apolipoprotein E	Binding to LDLR	Synthetic ApoE peptide
	CYP46A1	Activation	Efavirenz
	LXR receptor	Activation	LXR623 and gamitrinib
	SOAT1	Inhibition	shRNA
Fatty acid	SREBP-1 and SREBP-2	Inhibition	25-HC, fatostatin, and FGH10019
	Fatty acid oxidation	Inhibition	Etomoxir and anti-CD47 antibody
	Medium-chain acyl-CoA dehydrogenase	Inhibition	shRNA
	DGAT1	Inhibition	shRNA
Sphingolipid	SMPD1	Inhibition	Fluoxetine, pimozide, and loperamide
	N-acylsphingosine amidohydrolase 1	Inhibition	Carmofur
	GD2	Inhibition	Chimeric anti-GD2 dinutuximab beta
